# A perspective on essential components for adaptability and scalability to enhance equity-driven vaccination interventions: lessons from Mozambique and Malawi

**DOI:** 10.11604/pamj.supp.2025.51.1.47853

**Published:** 2025-06-11

**Authors:** Linda Shuro, Hanani Tabana, Emily Lawrence, Jocelyn Powelson, Gaspar Come, Lucia Knight, Helen Schneider

**Affiliations:** 1 School of Public Health, Faculty of Community and Health Sciences, University of the Western Cape, Robert Sobukwe Rd, Bellville, 7535, Cape Town, South Africa,; 2 VillageReach, 210 S Hudson St, Suite 307, Seattle, Washington 98134, USA,; 3 VillageReach, Maputo, Mozambique,; 4 School of Public Health & Family Medicine, Faculty of Health Sciences, University of Cape Town, Falmouth Building, Anzio Road, Observatory, 7925, Cape Town, South Africa,; 5 School of Public Health & SAMRC Health Services to Systems Research Unit, Faculty of Community and Health Sciences, University of the Western Cape, Cape Town, South Africa

**Keywords:** Routine immunisation, community-driven, scalability, equity

## Abstract

Global threats, including the COVID-19 pandemic, vaccine funding cuts, and persistent health inequities, are reversing decades of hard-won gains in immunisation coverage, particularly access for vulnerable populations. This highlights an urgent need to rethink how immunisation programs are designed and delivered, ensuring they are adaptable, scalable, equitable, and sustainable. Global partnerships such as Gavi, The Vaccine Alliance, United Nations Children´s Fund, and World Health Organization have been instrumental in strengthening immunisation systems and success hinges on approaches deeply rooted in local contexts. This perspective piece draws on the evaluation of the ‘Let´s Talk About Vaccines!’ project, which used participatory strategies to co-design and implement two co-created vaccination interventions in Malawi and Mozambique. It incorporates insights from Expanded Programme on Immunisation officials, global immunisation actors, and current literature. Key insights highlight the transformative potential of community-driven immunisation interventions that prioritize local ownership, inclusive governance, and tailored communication. Six essential components identified contributing to successful interventions include 1) Collaborative planning, design and implementation; 2) Innovative and culturally relevant communication modalities; 3) Gender responsive programming; 4) Strategic integration and alignment with health and community systems and global strategies; 5) Inclusive monitoring and feedback mechanisms; and 6) Continuous reflection and adaptation that balances fidelity with flexibility. We would argue that these guiding principles, adaptable and scalable, when embedded in immunisation strategies, can enhance equity and effectiveness across diverse contexts, aligning with global strategies such as Immunisation Agenda 2030, Big Catch Up, and GAVI 6.0. As Africa celebrates 50 years of immunisation progress, we must move beyond conventional models, adopting innovative strategies that amplify community voices, ensure flexibility in program design, and support national health priorities. The future of immunisation must embrace participatory design, local innovation, sustained commitment, and relentless focus on adaptability, ensuring no one is left behind amid shifting policies and limited resources.

## Perspectives

Since its launch in 1974, the World Health Organization´s (WHO) Expanded Programme on Immunisation (EPI) has been a cornerstone of global health efforts, driving unprecedented progress in vaccine access and coverage. The program has evolved over the past five decades to tackle emerging health threats. There´s no debate, vaccines save lives, a fact firmly supported by decades of rigorous scientific evidence. Yet, despite remarkable gains, persistent inequities, particularly in low- and middle-income countries (LMICs), continue to hinder universal vaccine access and uptake. Recent challenges, including the COVID-19 pandemic and funding cuts for vaccines, have further exacerbated barriers such as geographic inaccessibility, vaccine hesitancy, gender disparities, and weak health systems, limiting coverage [1-3].

Global immunisation efforts have averted millions of annual deaths [4]. In the past decades, we have seen that global partnerships have strengthened immunisation systems and expanded vaccine coverage [5]. For instance, key global immunisation frameworks and strategies such as the Immunisation Agenda 2030 (IA2030), GAVI 6.0 strategy, and the Big Catch-Up campaign have made significant improvements in access and coverage, emphasizing partnership-based, context-specific, innovative, equity-focused approaches that leverage data and community participation to close immunization gaps [6,7]. We have seen that despite recent funding cuts, there remains a commitment to improving vaccination efforts. However, while these global strategies provide strategic goals that prioritise equitable access, community engagement, and sustainable immunisation systems, their success hinges on how well they are adapted to local realities and contexts. Addressing existing challenges requires immunisation strategies that are both responsive to community needs and scalable across diverse settings [8]. Traditional top-down immunisation approaches often fail to achieve sustainable impact, as they may overlook the socio-cultural and behavioral dimensions of vaccine uptake [9].

We believe that addressing persistent coverage gaps requires more than just service expansion but demands a rethinking of how we engage under-vaccinated communities, how interventions are tailored to their specific needs, and how these efforts are embedded within existing health and social systems. In most African countries, Ministries of Health have made great strides in improving coverage. However, measles outbreaks in Malawi (and elsewhere) suggest immunity gaps, even if statistics look strong. We need to understand who is being left behind and why and include them in efforts to close the gap [6,10]. This highlights the need for innovative, context-specific solutions that integrate local expertise and priorities, that are not only effective but adaptable and resilient to evolving challenges to ensure no child is left behind [5,9]. This perspective piece draws on insights from the evaluation of the Let´s Talk About Vaccines! project, which used participatory strategies to co-design and implement vaccination interventions in Malawi and Mozambique [11-15], from EPI officials, conversations with actors in the global immunisation landscape and review of current literature from the immunisation ecosystem. The Let´s Talk About Vaccines! project in Mozambique and Malawi, comprised of interventions that were co-created with health officials, healthcare workers, caregivers, and local leaders to address context-specific barriers to childhood immunisation. In Mozambique, four integrated intervention components were implemented: (1) immunization education using co-designed pictorial cards, posters and empathetic messaging to improve caregiver knowledge and decision-making; (2) a mobile brigade prioritisation tool (matrix) for better outreach planning; (3) collaborative immunisation planning through monthly meetings between community focal points and health workers; and (4) supportive supervision from district EPI teams. These were reinforced by training, routine monitoring, and stakeholder engagement to ensure fidelity and local ownership. In Malawi, the intervention focused on three components: (1) community engagement and immunisation education through drama performances to address social and informational barriers; (2) the use of Amplio© talking books in health facilities to deliver immunisation messages, and (3) an adapted community and health facility score card approach, to strengthen outreach sessions and monitor progress.

Across both countries, mixed-methods evaluation approaches were applied in intervention and comparison sites to examine the reach, effectiveness, adoption, implementation, and maintenance of the interventions [11,12]. The project exemplifies the impact of community engagement in driving immunization success, addressing the social and behavioural dimensions of vaccine uptake, what we see as the “intangible software” of immunisation, including trust, motivation, and communication facilitating broader reach. Community-led projects such as these demonstrate the transformative potential of localised engagement, inclusive governance and tailored interventions, particularly in tackling vaccine hesitancy and improving immunisation coverage, especially in hard-to-reach areas. Based on the evaluation, literature, and our conversations, we have identified a set of critical implementation factors that we believe can be incorporated into immunisation programs at scale to make immunisation programming more localised and responsive to under-vaccinated communities´ needs and preferences and, ultimately, sustainably improve coverage. We argue that these components should serve as guiding principles in immunisation programming, ensuring that interventions are effective, equitable, and capable of expanding reach without compromising local relevance.

### Flexible and scalable essential components for advancing sustainable and equitable immunisation strategies

**Figure 1:**
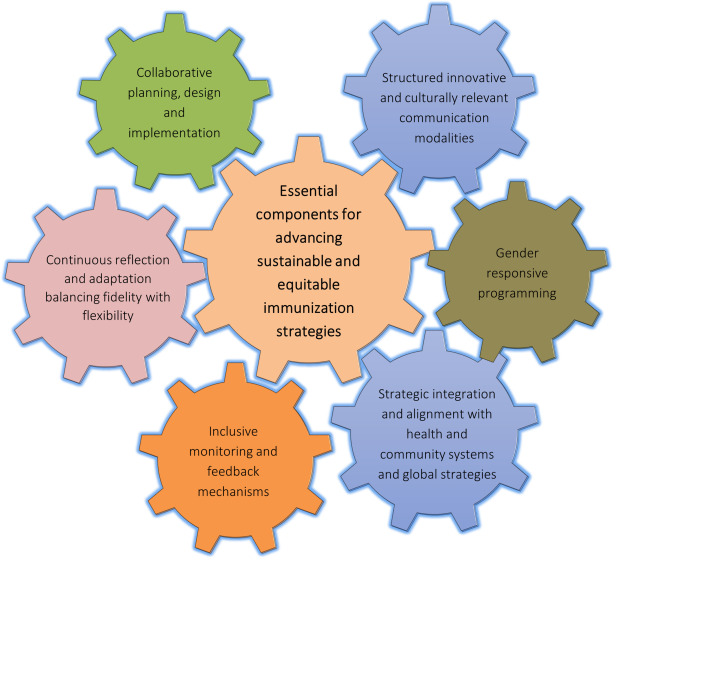
essential components for advancing sustainable and equitable immunisation strategies

We identified six adaptable and scalable essential implementation components that contribute to the success of immunisation efforts from the evaluation in Mozambique and Malawi and validated through literature and insights from EPI officials and conversations with actors in the global immunisation landscape. These components played a crucial role in enhancing community-responsive immunisation programming in both contexts, ultimately improving immunisation indicators such as coverage. Figure 1 illustrates the components, and each of these components is discussed further.

The components in Figure 1 function as interconnected cogwheels; the implementation of one drives the others. This illustrates a knock-on effect between these elements, reinforcing the idea that these components don´t operate in isolation. Instead, they exist within a continuous, dynamic process of reflection, adjustment, and adaptation, creating a cycle of mutual influence that is essential for driving equity-driven implementation in immunisation efforts.

### 1) Collaborative planning, design, and implementation

Collaborative planning and implementation means shifting from top-down approaches to shared decision-making that centres communities as equal partners. This involves intentionally engaging community members throughout the process, identifying challenges, co-designing solutions, and implementing strategies rooted in local realities, relationships, and leadership [12].

Participatory approaches, like **Community-Based Participatory Research (CBPR) and Human Centred Design (HCD)**, operationalise this by involving communities from the outset. They help reframe challenges into actionable strategies through lived experience, empathy, and shared power [14,15]. Establishing **collaborative implementation structures**, bringing together caregivers, health workers, officials, and local leaders, ensures continued engagement, strengthens trust, and fosters shared ownership. Empowering **local champions**, such as community health workers, drama group members, and caregiver representatives, is key to bridging the gap between health systems and communities. When trained and supported these trusted actors not only deliver messages but shape the immunisation agenda from within [14,15]. Their use of familiar formats like storytelling and peer dialogue helps embed vaccine messaging into daily life, address concerns authentically, and counter misinformation [16].

In practice, this was reflected in the Let´s Talk About Vaccines project, where **joint trainings and capacity-building workshops** built shared understanding, strengthened partnerships, and enhanced local leadership. These efforts supported both community-led advocacy and better coordination, leading to improved vaccination outcomes [17]. This collaborative, equity-driven approach aligns with global goals like Gavi 6.0, IA2030, and The Big Catch-Up. By embedding community voices and empowering local actors, immunisation programs become more trusted, resilient, and sustainable. Ultimately, collaborative implementation transforms immunisation from a health system directive into a shared social practice, essential for overcoming mistrust, improving uptake, and advancing health equity [7].

### 2) Structured, innovative and culturally relevant communication modalities

Effective immunization programming goes beyond delivering information, it requires communication modalities that are structured, participatory, innovative, and rooted in local culture and context. Top-down messaging often neglects local knowledge systems, cultural nuance, and systemic inequities contributing to hesitancy and mistrust [18,19]. In contrast, co-created and community-driven communication fosters dialogue, trust, and ownership [11,12,20]. These modalities are impactful not just because of their format, but because they are co-designed with communities, responsive to actual knowledge gaps, and embedded in existing cultural and social structures. Their design includes iterative feedback loops, enabling real-time adaptation and responsiveness.

In practice: **Malawi** used drama group performances and Talking Books tailored to local contexts. **Mozambique** introduced co-designed pictorial materials and empathetic, caregiver-informed messaging.

Across both countries, messages were shaped by caregivers´ expressed needs, increasing relevance and resonance. This approach aligns with global evidence showing that culturally embedded, community-driven strategies enhance health literacy, counter misinformation, and boost vaccine uptake. This reinforces a broader imperative, that communication in immunisation programs must be as dynamic and plural as the communities they serve. Our work emphasizes what others have articulated around the use of community-based strategies for effective communication to increase vaccine uptake [20]. While digital tools like Talking Books offer promise, they should complement, not replace, traditional, trusted formats such as community health talks. The takeaway: scaling equitable immunization requires not only technical solutions but human-centered communication that communities trust, relate to, and co-own. When designed this way, communication modalities become not just instruments of awareness, but enablers of transformation.

### 3) Gender responsive programming

Gender-responsive programming speaks to the design and delivery of immunisation interventions that actively recognize and address gender-based barriers, roles, and power dynamics. We know in many communities, especially in rural settings in Africa, deeply ingrained gender norms assign childcare and by extension, immunisation responsibilities almost exclusively to mothers. This not only overburdens women but also excludes men and extended family members from playing an active role in their children´s health [21]. The role of men in immunisation remains narrowly defined, often reduced to financial provision. This limited framing misses the opportunity to meaningfully engage men as active participants in their children’s health journeys. Gender responsive programming seeks to shift this paradigm and reimagines immunisation as a shared responsibility, not just a maternal task. It expands the role of men beyond the wallet, promoting their active participation in understanding vaccination schedules, supporting and even normalising their presence at clinic visits, and advocating for immunisation within the family and community. The goal is not to replace mothers´ roles but to distribute responsibility more equitably, making child health a shared, collective duty. When men are fully engaged, not only is the burden on mothers eased, but the entire immunisation ecosystem becomes stronger and more resilient.

The work in Mozambique and Malawi shows how intentionally structured community engagement and context-specific innovative strategies, such as targeted messaging on shared responsibility and leveraging extended family support, serve as key cogs in dismantling entrenched gender norms that traditionally assign childcare solely to women [11,12]. By reframing caregiving and immunisation as collective duties, these strategies invite men into conversations, create space for them to be seen not just as financial providers but as informed and active participants in their children’s vaccination journeys. This intentional male engagement triggers a knock-on effect, improving not just vaccination uptake but transforming the gender dynamics within families and communities. These inclusive strategies activate broader community structures, local leaders, grandmothers, and extended family members as vocal advocates for immunisation, further reinforcing social shifts toward gender equity. While positive trends in male engagement are emerging, entrenched gender norms, shaped in part by whether communities are matriarchal or patriarchal, remain a barrier in certain households. The visible increase in male engagement marks a critical step towards more inclusive systems. Gender-responsive programming is thus not only about increasing women´s participation in immunisation but about creating inclusive systems that recognize and address gender disparities, engage all caregivers, address power dynamics, and ensure equitable access to vaccines.

### 4) Strategic integration and alignment with health and community systems and global strategies

Strategic integration and alignment mean embedding immunisation interventions within existing health and community systems, policies, and global strategies. Rather than operating as standalone or parallel initiatives, integrated programs are designed to work with and through established structures promoting coherence, reducing duplication, and fostering long-term sustainability. The experiences in Malawi and Mozambique show that aligning with local and national strategies, such as the mother care groups and Malawi Community Health Strategy in Malawi and Reach Every District Reach Every Child (REDREC) in Mozambique, not only fosters community trust and resource mobilization but also strengthens the institutional foundations needed for long-term adoption [22,23]. This integration supports behavior change, improves service delivery, and ensures that immunisation initiatives are responsive to local priorities while contributing to broader development agendas. However, it also emphasises the need for ongoing coordination across health sectors to avoid fragmentation and maximize system-wide efficiency. Efforts in both contexts show that immunisation interventions should not only be rooted in national strategies but also reflect a broader commitment to global immunisation agendas championed by organizations such as WHO, UNICEF, and GAVI.

Strategic integration is not just a technical process, it is a foundational enabler of equity and sustainability. Working within systems, rather than around them, we increase the likelihood that interventions will be adopted, scaled, and maintained, ensuring effective immunisation efforts positioned to continue reaching vulnerable populations, especially those furthest behind, well into the future.

### 5) Inclusive monitoring and feedback mechanisms to inform decision-making

Inclusive monitoring and feedback mechanisms, when paired with leveraging routine health information systems data, are critical for ensuring that data down to the community level is used for informed decision making within immunisation programs. Inclusive monitoring and feedback mechanisms involve creating space for community voices through structured feedback loops and participatory approaches to enhance both the legitimacy and responsiveness of interventions, enabling real-time course correction and long-term scalability. When monitoring is designed to be participatory, it transforms communities from passive recipients into active contributors, allowing for real-time learning, stronger accountability, and deeper program legitimacy. We´ve seen through the Let´s Talk About Vaccines project and literature that these inclusive mechanisms led to greater community ownership, more context-sensitive communication strategies, and improved targeting of resources. In Mozambique, innovations such as monitoring dashboards tracking under-two immunisation targets, WhatsApp platforms for real-time coordination, monthly facility check-ins, and caregiver feedback platforms enable a 360-degree view of implementation realities. In Malawi, similarly, layered strategies ranging from dashboards and digital communications to structured community feedback loops and coordination with the District Health Office ensures that insights flow vertically (from communities to national level) and horizontally (across implementation partners), aligning interventions with both local priorities and national strategies.

The real value of inclusive monitoring and feedback mechanisms lies in their ability to drive adaptive, community-responsive programming. By creating space for community voices, they strengthen collaborative planning, build trust, and increase ownership. When feedback loops become routine, they not only track progress but trigger meaningful adaptations rooted in lived experience. Tools like post-performance surveys, Talking Books, and scorecards capture user perspectives beyond numbers, ensuring shifts are both data-informed and context-sensitive. These mechanisms act as strategic enablers that keep immunisation programming equitable, resilient, and truly people-centred.

While inclusive monitoring and feedback mechanisms can greatly improve decision-making, a critical gap that remains to be addressed in many LMICs is the poor quality and use of routine health information systems data. Strengthening this data through integration of data systems across departments, investing in robust data infrastructure and tools, strengthening supportive supervision, training data collectors, and improving data visibility can complement community-level insights to inform the design and delivery of equitable and inclusive immunization services [24-26] As the WHO emphasizes, data-driven approaches enable health systems to close immunisation gaps with precision [24].

### 6) Continuous reflection and adaptation: balancing fidelity with flexibility in the implementation of immunisation strategies

Achieving lasting impact in immunisation programming requires **a continuous process of reflection and adaptation** that balances fidelity to evidence-based strategies or protocols with flexibility to respond to contextual realities. Whilst it goes without saying, a strong commitment to maintaining fidelity with the use of agreed upon Standards of Practices (SOPs) is important, at the same time, flexibility is critical to navigating barriers. The participatory nature of interventions fosters a culture of adaptive learning and incorporation of feedback from communities, refinement of tools, and delivery strategies adjusted to optimize relevance and reach. Even when resource limitations require abridged implementation, stakeholders work collaboratively to uphold the integrity and effectiveness of the interventions [11,12].

Experiences from Malawi and Mozambique highlight that fidelity and flexibility are not mutually exclusive. Rather, their balance is essential for sustainable, equity-driven immunisation programs that can scale effectively across diverse settings. Moreover, the synergy between data systems and community engagement feeds into a continuous process of reflection and adaptation, the very heart of implementation equity. Embedding adaptive processes within program design strengthens resilience, fosters local ownership, and enhances the ability to respond to emerging challenges without compromising quality [27].

These suggested essential components above act as interlocking mechanisms, where movement in one initiates motion in the others, i.e., inclusive monitoring and feedback mechanisms, promote reflection, and allow for timely adjustments based on community input and implementation challenges.

## Conclusion

Reflecting on the vital lessons from the Let´s Talk About vaccines project, reinforced by current literature and conversations, it is increasingly clear that the future of immunisation must prioritise adaptability, scalability, and deep community engagement. Traditional, top-down approaches are no longer sufficient to meet the complex and evolving needs of today´s populations, especially those most vulnerable. By incorporating the guiding principles highlighted in this perspective piece, such as culturally sensitive communication, we can foster immunisation programs that are not only effective but also resilient in the face of emerging challenges. These principles also align and reinforce global strategies that call for equity-focused, scalable solutions. Moreover, the essential components we highlight show strong potential for adaptation across other health priorities and geographies, supporting the development of equity-driven solutions. As we mark 50 years of immunisation efforts in Africa, we must commit to strategies that amplify community voices and ensure that programmes are flexible enough to adapt to changing contexts. This requires a shift in mindset from paternalistic approaches to collaborative partnerships that empower local stakeholders and integrate into existing health and community systems. Sustainability lies not in uniform solutions, but in dynamic interventions that adapt and evolve with the communities they serve. The impact of this commitment to equity was evident in improved immunisation coverage in Malawi and Mozambique in the intervention sites, which demonstrates the power of this approach. In a post-pandemic world, where global health threats persist and vaccine inequities remain stark, we must commit to a new era, one defined by participatory design, local innovation, policy integration, and relentless focus on leaving no one behind. By embracing the essential cross-contextual components proposed in this perspective piece, we can better navigate barriers to vaccine uptake, deepen trust, and ensure immunisation programs are not only effective but are truly equity-driven and also resilient to shifting policy landscape and resource constraints.
